# Flexible Graphene Film-Based Antenna Sensor for Large Strain Monitoring of Steel Structures

**DOI:** 10.3390/s24134388

**Published:** 2024-07-06

**Authors:** Shun Weng, Jingqi Zhang, Ke Gao, Hongping Zhu, Tingjun Peng

**Affiliations:** 1 School of Civil and Hydraulic Engineering, Huazhong University of Science and Technology, Wuhan 430074, China; wengshun@hust.edu.cn (S.W.); hpzhu@mail.hust.edu.cn (H.Z.); m202271486@hust.edu.cn (T.P.); 2 Hubei Key Laboratory of Control Structure, Huazhong University of Science and Technology, Wuhan 430074, China

**Keywords:** strain sensors, patch antenna, graphene film, large strain monitoring, dual-resonant

## Abstract

In the field of wireless strain monitoring, it is difficult for the traditional metal-made antenna sensor to conform well with steel structures and monitor large strain deformation. To solve this problem, this study proposes a flexible antenna strain sensor based on a ductile graphene film, which features a 6.7% elongation at break and flexibility due to the microscopic wrinkle structure and layered stacking structure of the graphene film. Because of the use of eccentric embedding in the feeding form, the sensor can be miniaturized and can simultaneously monitor strain in two directions. The sensing mechanism of the antenna is analyzed using a void model, and an antenna is designed based on operating frequencies of 3 GHz and 3.5 GHz. The embedding size is optimized using a Smith chart and impedance matching principle. Both the simulation and experimental results verify that the resonant frequency and strain magnitude are linearly inversely proportional. The experimental results show that the strain sensitivity is 1.752 kHz/με along the geometric length and 1.780 kHz/με along the width, with correlation coefficients of 0.99173 and 0.99295, respectively.

## 1. Introduction

Steel structures, which are extensively utilized in civil infrastructure [1], are vulnerable to material degradation due to sustained loading and environmental influences [2]. Potential structural damage or failure may be heightened when monitoring and maintenance are not conducted promptly [3]. In the field of structural health monitoring (SHM), strain has been identified as a crucial indicator for providing insight into the health of steel structures under loads and ambient factors [4,5,6,7]; hence, monitoring structural strain is one of the most important issues in the SHM of steel structures. Traditional strain-monitoring technology mostly adopts vibrating wire strain gauge [8], optical fiber strain gauge [9] and metal foil strain gauge [10]. Other methods involve resistive strain gauge [11], a Carlson strain meter [9], fiber Bragg grating [12,13], sensing skins [14,15], and digital image correlation [16]. As strain sensing technology has advanced, traditional strain sensors have demonstrated significant stability, yet they retain certain limitations [9]. For instance, fiber optic-based sensors can transmit data via optical fibers but necessitate cable deployment for power supply, thereby constraining their applicability and potentially complicating installation in some scenarios [12,13]. In the realm of contemporary strain sensing research, wireless passive technology has emerged as a pivotal area of focus [17]. This approach offers the potential for power and data transmission without the need for cables, thereby enhancing installation flexibility and convenience [18]. Nonetheless, this technology remains in developmental phases and may encounter challenges pertaining to cost, stability, and the accuracy of data [17,18].

Moreover, steel is a ductile material; thus, the steel structure tends to have a large yield strain or fatigue creep before cracks occur [19]. Therefore, monitoring large strain in steel structures is very important for safety [20]. Nevertheless, the effective ranges of traditional strain sensors are less than the plastic and yield deformation range of steel, making it difficult to meet the needs of large strain monitoring of steel structures [21,22]. Furthermore, these technologies are based on active wired sensors, which require extensive cabling for power and signal transmission, thereby increasing installation and maintenance costs [23]. Thus, there is an urgent need to develop a wireless, passive, and real-time monitoring system capable of detecting large strain in steel structures.

Microstrip antennas offer a promising solution due to their ability to operate as passive wireless sensors. These antennas experience a shift in their resonant frequency when their geometric dimensions change, a property that can be exploited for strain sensing [24]. The linear relationship between the resonant frequency shift and the antenna’s dimensions allows microstrip antennas to serve as strain sensors when attached to a structure [21]. As the structure deforms, the antenna’s dimensions change, leading to a measurable shift in the resonant frequency, which can be detected using a vector network analyzer (VNA) [23].

Xie et al. [21] proposed an RT-5880 rectangular patch antenna center-fed strain sensor based on the relationship between resonant frequency offset and strain, which is used to monitor structural strain. The sensor showed a strain range of 700 με in the experiment, with a sensitivity of 2.413 kHz/με. Moreover, the influence of antenna length and width direction deformation on sensitivity was studied through numerical simulation and experiments. The results showed that the influence of width direction deformation on sensitivity can be ignored, while the change in resonant frequency has a good linear relationship with the strain in the direction of antenna length. Huang et al. [4] established a theoretical relationship between antenna resonant frequency variation and strain and temperature variation and designed a rectangular eccentric-fed antenna sensor using Rogers RT/duroid 5880 as the substrate. Thermomechanical testing was conducted on it to verify the theoretical prediction. The experimental strain varied from 0 to 355 με. For TM_10_ mode and TM_01_ mode, the measured strain sensitivities were 0.74 ppm/με and 0.43 ppm/με (i.e., about 3.774 kHz/με and 2.580 kHz/με), respectively. This indicated that the normalized resonant frequency variation of the antenna sensor is linearly related to the applied strain and temperature variation.

However, current microstrip antenna sensors, which are often made of metal, are rigid and do not conform well to steel structures, particularly those with irregular shapes. Although a traditional metal antenna can achieve high gain, with its range, it becomes difficult to perceive large deformation [25]. This limits their effectiveness in monitoring large strain in such structures [26]. Therefore, there is a need for a flexible conductive material with which to fabricate microstrip antenna sensors that can closely adhere to the structural surface. Graphene, with its exceptional electrical, mechanical, and thermal properties, has emerged as a promising material for flexible sensor devices [27]. This paper presents the design and analysis of a graphene film antenna sensor, which, compared to traditional copper antennas, offers enhanced corrosion resistance and flexibility due to its micro-wrinkle structure [28]. This makes graphene film antenna sensors well-suited to large strain monitoring of steel structures with varying geometries.

The study begins with an examination of the material characteristics and sensing performance of the graphene film antenna strain sensor. Using the electromagnetic simulation software Ansys HFSS 2018, the antenna’s radiating patch dimensions were optimized to achieve an S_11_ parameter peak below −10 dB. The impedance matching was confirmed through Smith chart analysis and characteristic impedance metrics, resulting in a proposed dimensional scheme for the antenna sensor. Subsequently, the strain sensing performance of the graphene antenna was simulated within a range from 0 to 65,000 µε using HFSS, revealing the strain sensing sensitivity in two orthogonal directions. The strain sensing sensitivities in the length and width direction in the range from 30 to 522 με were verified through experiments to be 1.751 kHz/με and 1.780 kHz/με, respectively, being of a similar order of magnitude as the above traditional metal antenna sensors. These findings confirm the feasibility of the designed graphene patch antenna for monitoring large strains in steel structures.

## 2. Strain Monitoring Principle and Antenna Design

### 2.1. Conductivity and Large-Scale Properties of Graphene Films

**Figure 1:**
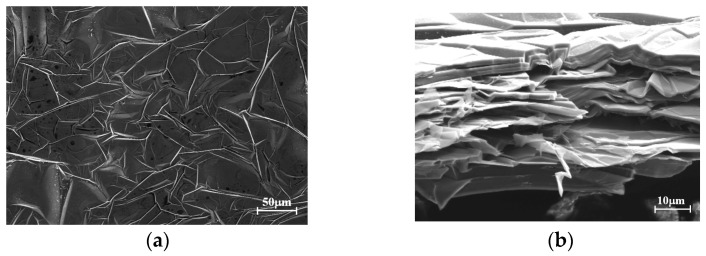
Morphological diagram of graphene film. (**a**) In-plane micro-wrinkle structure of graphene film. (**b**) Out-of-plane layered stacking structure of graphene film.

In recent years, graphene film has been widely studied and applied due to its flexibility, light weight, high conductivity and excellent chemical stability [29,30]. Graphene film has good conductivity, excellent flexibility, light weight, high thermal conductivity and corrosion resistance. Therefore, it has unique advantages in the application of RF microwave antenna in large deformation sensing. The conductivity of the graphene film developed by He et al. can reach 1.1 × 10^6^ S/m, which is lower than the conductivity of copper at 1.32 × 10^7^ S/m, but its performance in antenna applications is comparable to that of copper [31]. Graphene film has good mechanical stability and can be bent 1000 times without changing its resistance [1], and the surface of the film remains flat after multiple folds [2]. In addition, graphene film has good chemical stability. Under the same conditions of salt spray treatment, graphene films have better corrosion resistance than copper. When copper rusts, there is no significant change in the surface of graphene films [3]. In addition, its elongation at break is as high as 6.7% (67,000 με), meaning it can meet the working requirements of large strain sensing. The graphene film used in this paper has a conductivity of 10^6^ S/m, meaning it can be used to make antennas. The microstructure under the electron microscope is shown in Figure 1. Figure 1a shows that there are a large number of micro-folds on the surface of the graphene film. The micro-folds expand with tensile deformation, hence the film has good extensibility. Figure 1b shows the cross-sectional microstructure of the graphene film. It is observed that the graphene film is stacked with multilayer graphene nanosheets, and the relative slip among the layers also improves the ductility of the film [32]. Its microstructure determines that graphene patches naturally have a large range of properties.

### 2.2. Principle of Bidirectional Strain Monitoring

**Figure 2:**
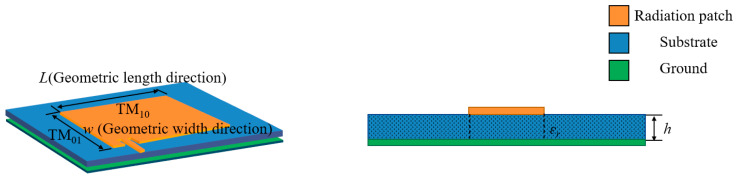
Hierarchical structure model of flexible graphene antenna sensor.

The flexible antenna consists of a graphene radiation patch, a dielectric substrate, and a conductive ground plate. The layered structure model is shown in Figure 2.

According to the cavity model theory of microstrip antenna [33], the space between the radiation patch and the ground plate is regarded as the upper and lower electric walls, and the surrounding is the leakage wave space of the magnetic wall. The resonant frequency *f_mn_* operating in the TM*_mn_* mode is obtained as shown in Formula (1). In order to simultaneously excite the radiation of the patch antenna along the length and width directions, an eccentrically fed patch is used to monitor the strain in both directions. The eccentric feed patch is mainly radiated by two basic resonant modes of TM_01_ and TM_10_. The TM_01_ radiation mode is parallel to the geometric length direction of the radiation patch, corresponding to the resonant frequency *f*_01_. The TM_10_ radiation mode is parallel to the geometric width direction of the radiation patch, corresponding to the resonant frequency *f*_10_. In terms of *f*_01_, the relationship between the resonant frequency of the eccentrically fed antenna and the geometric length *l* of the patch is shown in Formula (2).

(1) $$f_{mn}=\frac{c}{2\sqrt{\varepsilon_{e}}}\sqrt{{\left(\frac{m}{w+2\Delta w}\right)}^{2}+{\left(\frac{n}{l+2\Delta l}\right)}^{2}}$$

where each group of *m* and *n* represents a mode satisfying the cavity boundary condition, and *f_mn_* is the resonant frequency corresponding to the TM*_mn_* mode of the antenna. The offset rectangular microstrip antenna usually works in TM_01_ and TM_10_ modes, so this study takes two sets of (*m*, *n*) values as (0, 1) and (1, 0), respectively. c is the speed of light, and *ε_e_* is the effective dielectric constant. *l* and *w* are, respectively, the geometric length and width of the radiation patch; Δ*l* and Δ*w* are the corresponding extension length of *l* and *w* caused by the edge effect.

(2) $$f_{01}=\frac{c}{2\sqrt{\varepsilon_{e}}}\frac{1}{l+2\Delta l}$$

The calculation formula of *ε_e_* is

(3) $$\varepsilon_{e}=\frac{\varepsilon_{r}+1}{2}+\frac{\varepsilon_{r}-1}{2}{\left(1+12\frac{h}{w}\right)}^{\frac{1}{2}}$$

where *ε_r_* is the relative dielectric constant of the dielectric, and *h* is the thickness of the dielectric substrate.

The calculation formula of Δ*l* is

(4) $$\Delta l=0.412h\frac{\left(\varepsilon_{e}+0.3)(w/h+0.264\right)}{\left(\varepsilon_{e}-0.258)(w/h+0.8\right)}$$

**Figure 3:**
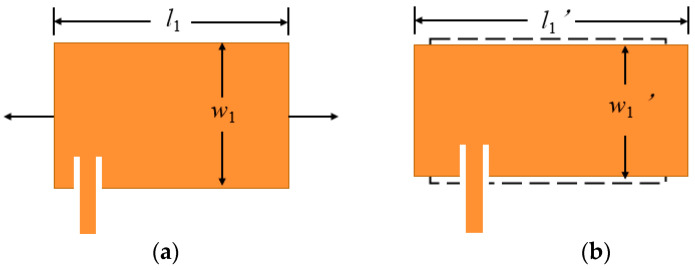
The influence of stress on the size of rectangular patch antenna. (**a**) Size diagram of radiation patch before stress. (**b**) After the radiation patch is subjected to stress.

It can be seen from Formulas (2) and (4) that when the size of the radiation patch deforms with the structure, its geometric length and extension length *l* and Δ*l* will change, resulting in a change in the resonant frequency. As shown in Figure 3, assuming that the Poisson’s ratio *v_P_* of the radiation element is equal to the Poisson’s ratio vs. of the dielectric substrate, when the rectangular patch antenna generates strain *ε_l_* in the length direction, the ratio *w*/*h* of the width and thickness *h* will remain unchanged, and the effective dielectric constant *ε_e_* will not be affected by the strain *ε_l_*.

Taking the deformation in the geometric length direction as an example, the first-order resonant frequency is shown in Formula (5) after the strain *ε_l_* occurs.

(5) $${f_{01}}^{\prime }=\frac{c}{2\sqrt{\varepsilon_{e}}}\frac{1}{l+2\Delta l+\varepsilon_{l}\cdot l}$$

The variation of the resonant frequency divided by the absolute value of the strain is defined as the strain sensing sensitivity *k*, as in Formula (6):

(6) $$k=\frac{{f_{01}}^{\prime }-f_{01}}{\varepsilon_{l}}=-\frac{f_{01}}{1+2\Delta l/l+\varepsilon_{l}}$$

The tensile strain *ε_l_* > 0. The absolute value of sensitivity *k* is positively correlated with the initial resonance frequency in the corresponding direction, i.e., the higher the initial resonance frequency, the greater the absolute value of the strain sensing sensitivity. In addition, the absolute value of the strain sensing sensitivity is less than the initial resonance frequency because the denominator in Equation (6) is greater than 1.

### 2.3. Antenna Size Optimization Design

In this paper, the inset feeding method [24] is selected. Because of its special shape, it will affect the electrical length and electrical width of the antenna patch, which will affect the initial resonance frequency of the antenna and the strain sensing sensitivity of the antenna. Therefore, the embedding position of the antenna is optimized.

According to Formulas (2)–(4), the corresponding relationship between the resonant frequency and the geometric size can be calculated after the operating frequencies in two directions are selected. The geometric size of the antenna radiation patch can be calculated, and the preliminary size design of the antenna can be carried out accordingly. The initial operating frequencies selected in this paper are 3 GHz and 3.5 GHz, respectively, which show potential for wireless sensing via 5 G communication [34,35]. As a result, the geometric length and geometric width of the radiation patch are calculated to be 30.005 mm and 24.663 mm through Formula (2), and this study takes integers as 30 mm and 25 mm.

**Figure 4:**
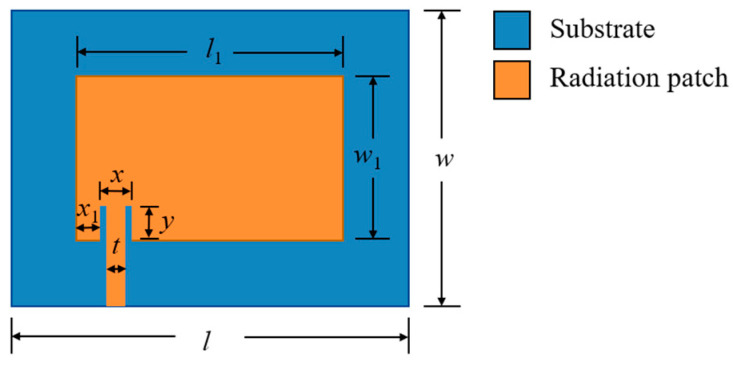
Size schematic of patch antenna.

The resonant frequency *f_mn_* and the return loss S_11_ are two important parameters of the microstrip patch sensor. The return loss parameter S_11_ is an important parameter for evaluating the impedance matching of the antenna. According to Xie et al. [36], an S_11_ value below −10 dB indicates good impedance matching between the antenna and transmission line, which means that the signal can be effectively transmitted from the transmission line to the antenna, and very little signal will be reflected back to the source. In this paper, the eccentric feed mode is adopted. This is because the position deviation of the feed point from the center can simultaneously excite the two basic resonant modes (TM_01_ and TM_10_) to monitor the strain along the length and width directions. An inset feed is adopted to meet the needs of antenna miniaturization. In this case, in order to achieve the optimization goal of a peak impedance matching value, S_11_, of less than −10 dB, high-frequency electromagnetic simulation software (HFSS) is used to optimize the embedded size of the feed-line, i.e., *x*_1_, *x*, *t* and *y* in Figure 4. *l*_1_ and *w*_1_ represent the dimensions of the patch, while *w* and *l* denote the dimensions of the substrate.

**Figure 5:**
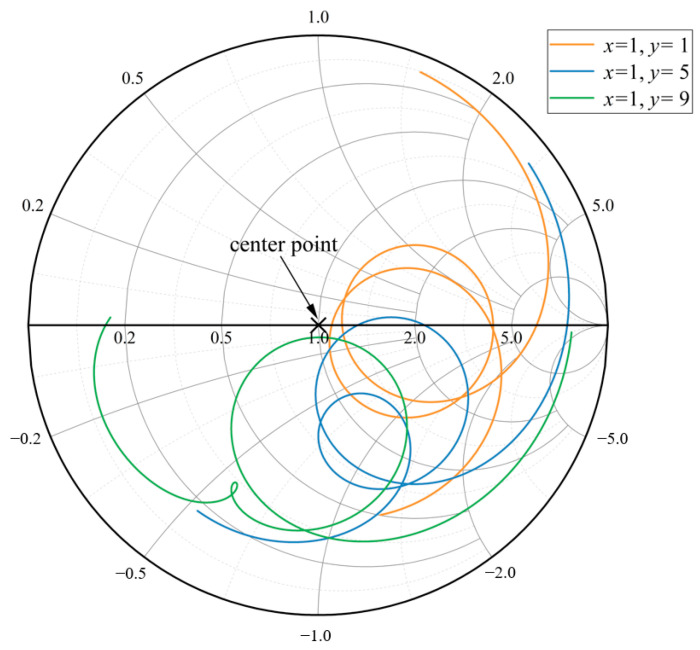
Smith chart corresponding to *x*_1_ = 1, *y* = 1,5,9.

Firstly, the Smith chart is used to analyze the size of the embedded feeder. The Smith chart is a visual tool which can reflect the impedance matching of the antenna in the complex impedance matching of the RF circuit [37]. This paper analyzes the approximate range of the embedded size based on the Smith charts. As shown in Figure 5, the Smith chart is composed of an equal-resistance circle system and an equal-reactance circle system. The Smith circle adopts normalized impedance, and the center point represents the system impedance. When the intersection point of the Smith circle diagram and the transverse coordinate axis corresponding to a certain frequency of the antenna is close to the center of the circle, it indicates good impedance matching of the antenna at that frequency, and therefore it meets the requirements. For instance, Figure 5 illustrates three typical cases of the Smith chart in different embedding conditions, namely the cases of no intersection with the resistance axis, one intersection, and two intersections. To facilitate discrimination, two intersections of the resistance axis are selected for analysis. The embedding position *x*_1_ is taken from 1 mm to 7 mm, and the embedding depth *y* is taken from 1 mm to 3 mm.

The corresponding intersection coordinates of the Smith chart and the horizontal axis are listed in Table 1. When *x*_1_ is in the range of 1 to 4 mm and *y* is in the range of 1 to 2 mm, the normalized resistances of the two resonant frequencies are close to 1, that is, the impedance matches well. As the value of *x*_1_ increases, the difference between the normalized resistances of the two intersections and the coordinate axis also increases from 1, meaning the impedance matching become worse. Therefore, it is preliminarily estimated that 1 mm ≤ *x*_1_ ≤ 4 mm and 1 mm ≤ *y* ≤ 2 mm are proper ranges for the embedded feeder.

**Figure 6:**
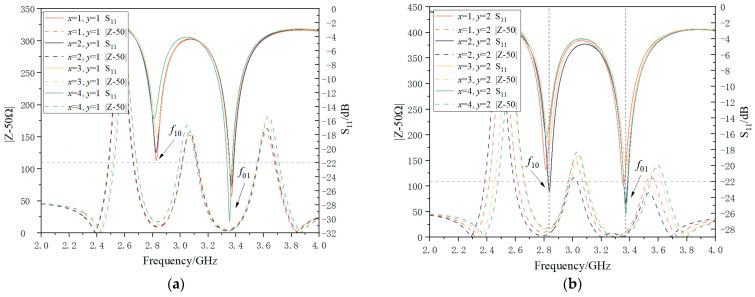
The input impedance and S_11_ curve of the antenna corresponding to different feed positions. (**a**) The input impedance and S_11_ curve of the corresponding antenna when the feed depth *y* = 1, *x*_1_ = 1, 2, 3, 4. (**b**) The input impedance and S_11_ curve of the corresponding antenna when the feed depth *y* = 2, *x*_1_ = 1, 2, 3, 4.

Moreover, as shown in Figure 6, the optimal feeder embedding size is further determined by taking the absolute value after the difference between the antenna impedance and the standard impedance of the transmission line 50 Ω. If the difference is 0, the antenna impedance matching is good. Therefore, *x*_1_ = 2 mm and *y* = 2 mm are selected as the embedding dimensions of the antenna. Compared to traditional design, the optimization design using Smith chart diagram and impedance matching principle is more visual and can select the relatively optimal size scheme within a certain range. According to Figure 6a,b, only the size scheme with *x*_1_ = 2 mm and *y* = 2 mm can achieve peak values of less than −22 dB in both resonance modes, indicating good impedance matching.

**Figure 7:**
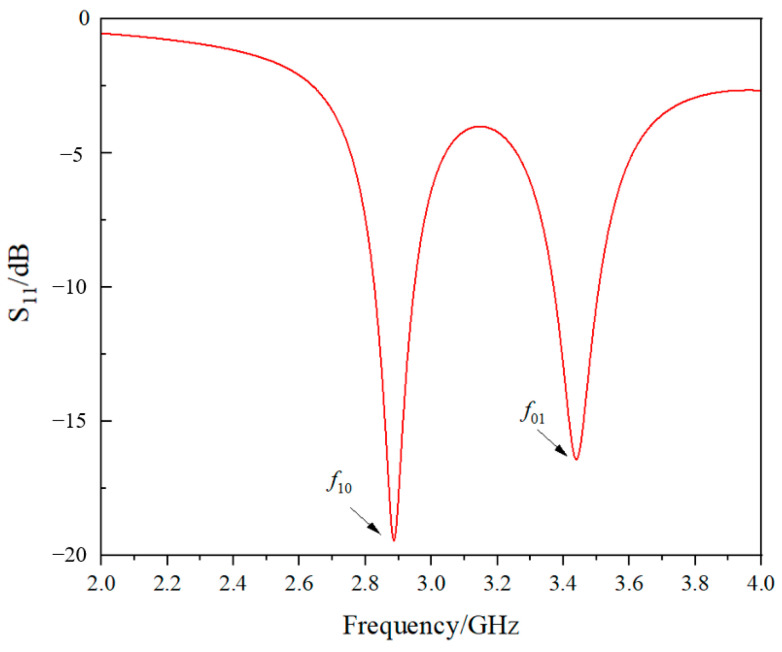
The return loss curve corresponding to the embedding position *x*_1_ = 2 mm, *y* = 2 mm.

At the same time, polyethylene terephthalate (PET) material is selected as the dielectric substrate of the antenna. Its relative dielectric constant is 3.0, and the thickness is 0.35 mm. The initial size is calculated from the Formulas (1)–(4), and then the size is adjusted to meet the impedance matching requirement that the S_11_ value should be less than −10 dB. A set of eccentric feed antenna size design schemes are given in Table 2 below. The corresponding return loss curve is shown in Figure 7. The resonant frequencies along the length and width directions are 2.887 GHz and 3.440 GHz, respectively. Correspondingly, the peak values of S_11_ along the length and width directions are less than −10 dB.

## 3. Simulation of Strain Sensing Performance of the Antenna Sensor

### 3.1. Modeling of the Antenna

**Figure 8:**
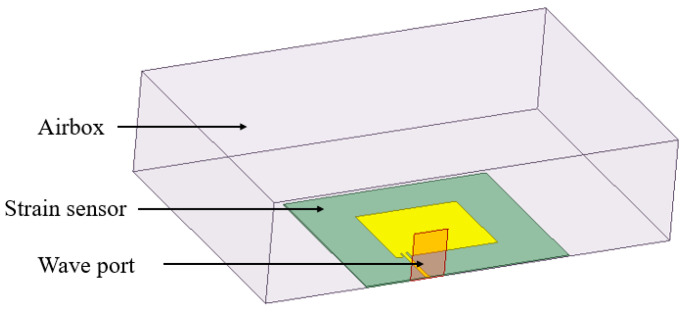
HFSS modeling image when the embedding position *x* = 2 mm, *y* = 2 mm.

Based on the size given in Table 2, the model obtained by HFSS finite element simulation modeling is shown in Figure 8. The substrate material is PET, the relative dielectric constant is 3.0, the dielectric loss angle tangent is 0.06, and the size is 50 mm × 60 mm × 0.35 mm. The thickness of the graphene film is 28 μm [35], which is much smaller than the substrate thickness of 0.35 mm. For convenient calculation, the radiation patch is set to a two-dimensional shape and Perfect E boundary [36]. The size of the upper radiation patch is 25 mm × 30 mm, and the size of the lower radiation patch (ground) is 50 mm × 60 mm, which is the same as the plane size of the substrate. The elongation at break of the selected graphene film is 6.7% (67,000 µε) [31]. In this simulation, the strain range is set to two working conditions: (1) a small strain condition from 0 to 600 µε, with a fixed interval of 100 µε for each analysis step, and (2) large strain condition from 0 to 65,000 µε, with a fixed interval of 5000 µε.

### 3.2. Simulation Results

**Figure 9:**
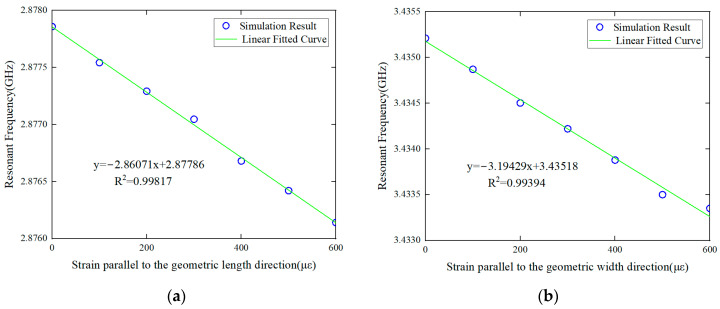
Strain–resonance frequency mapping relationship in the small strain range (**a**) along the geometric length direction and (**b**) along the geometric width direction.

In the small strain condition, the relationship between the antenna strain and the resonant frequency is shown in Figure 9. It is observed that there is a linear correlation between the strain and the resonant frequency, which is consistent with the theoretical derivation of Formula (5). As shown in Figure 9a, the initial resonance frequency of the antenna sensor in the geometric length direction is 2.878 GHz, and the strain sensing sensitivity is 2.861 kHz/με with a high R^2^ value of 0.998. This means each 1 με tensile strain along the length direction will cause the resonance peak f_10_ to decrease by 2.861 kHz. As shown in Figure 9b, the resonant frequency of the antenna in the geometric width direction is 3.435 GHz, and the strain sensing sensitivity is 3.194 kHz/με, with a high R^2^ value over 0.994. That is, each 1 με tensile strain along the width direction will cause the resonant peak f_01_ to decrease by 3.194 kHz.

**Figure 10:**
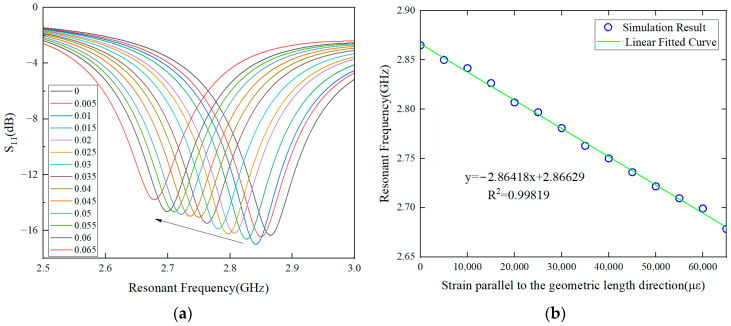
Strain–frequency shift relationship in the large range along length direction. (**a**) Strain–frequency mapping relationship. (**b**) Strain–resonance frequency mapping relationship.

**Figure 11:**
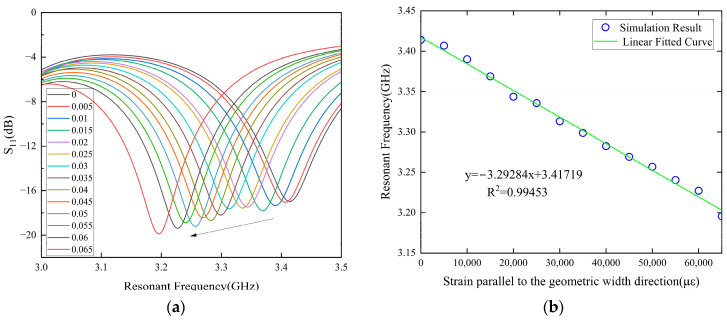
Strain–frequency shift relationship in the large range along the width direction. (**a**) Strain–frequency mapping relationship. (**b**) Strain–resonance frequency mapping relationship.

In the large strain condition, the strain–resonance frequency mapping relationship of the antenna along the length and width directions is shown in Figure 10 and Figure 11. The initial resonance frequency in the length direction is 2.866 GHz, and the corresponding strain sensing sensitivity is 2.864 kHz/με. The initial resonant frequency in the width direction is 3.417 GHz, and the strain sensing sensitivity is 3.293 kHz/με.

Comparing the results of the two working conditions, it can be seen that the tensile strain sensing sensitivity of the antenna is slightly smaller than its initial resonance frequency, which is consistent with the conclusion of Formula (6). The strain sensing sensitivity in the width direction of the antenna is larger than that in the length direction. This is because the initial resonant frequency is inversely proportional to the geometric size (shown in Formula (4)), and the strain sensing sensitivity is proportional to the initial resonant frequency (shown in Formula (6)). Therefore, the geometric size is inversely proportional to the strain sensing sensitivity. In addition, according to Table 3, the strain sensitivity under small strain condition is slightly different from that under large strain condition. Considering the variation of the sensitivities is less than 3% of their baseline values, the slight differences can be ignored. As a result, the strain sensitivity of the designed graphene patch antenna along the length direction and the width direction is about 2.86 kHz/με and 3.29 kHz/με, respectively.

## 4. Strain Measurement Experiment

**Figure 12:**
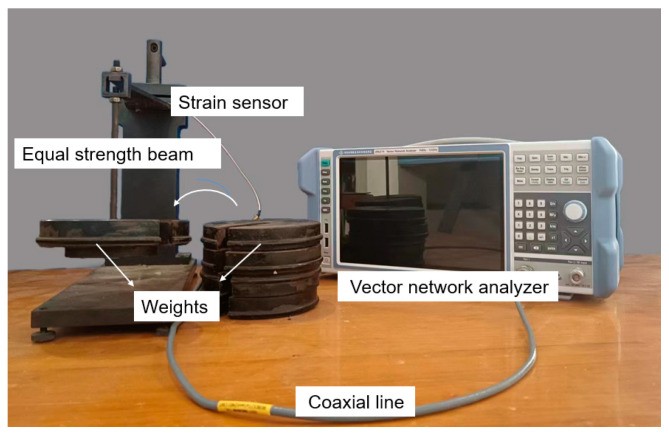
Test equipment connection diagram.

**Figure 13:**
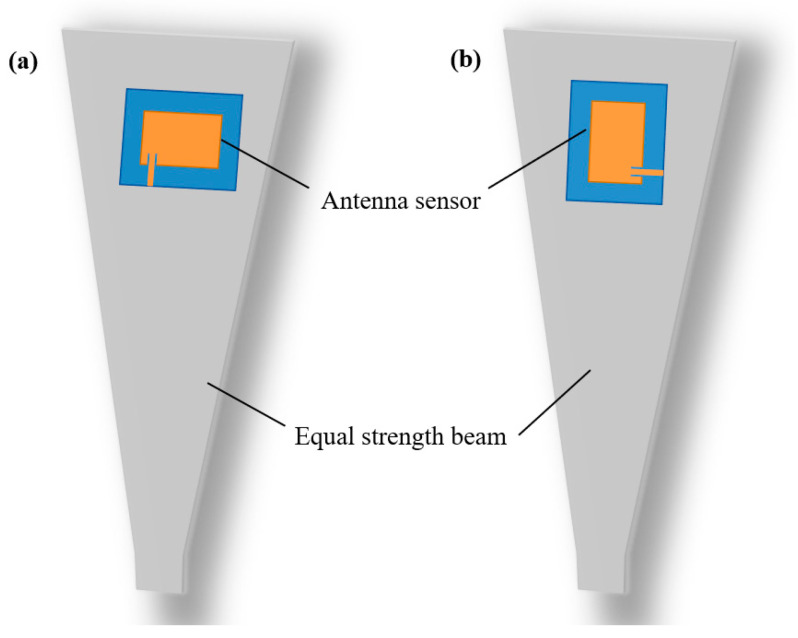
Strain measured in two directions with different pasting directions. (**a**) Strain along geometric width. (**b**) Strain along geometric length.

Figure 12 illustrates the setup of the strain sensing experiment. The microstrip antenna was bonded to the PET dielectric substrate with spray glue. The PET substrate was bonded to the equal-strength beam with strong glue. Figure 13a shows the adhesive position of the antenna sensor when measuring strain along the geometric width direction, and Figure 13b shows the adhesive position of the sensor when measuring strain along the geometric length direction. The equal-strength beam adopts a variable cross-section, and when a force F is applied at the free end, the strain generated at each part of the beam is equal, which facilitates the pasting of metal foil gauges and sensors. The feedback line of the antenna was connected to the vector network analyzer (VNA) by a coaxial line. The deformation of the equal-strength beam was caused by adding weights to its free end. There were five weights that caused the equal-strength beam to generate strain from 0 to 500 με. The antenna size was changed with the beam’s deformation, and thus the resonance frequency of the antenna sensor drifted. The VNA we selected is ZNLE 14 from Rohde & Schwarz (Vimperk, Czech Republic). Its operating frequency is from 1 MHz to 14 GHz, and its frequency resolution is 1 Hz. Its performance can meet the needs of strain monitoring in the experiment.

### 4.1. Experimental Process

**Figure 14:**
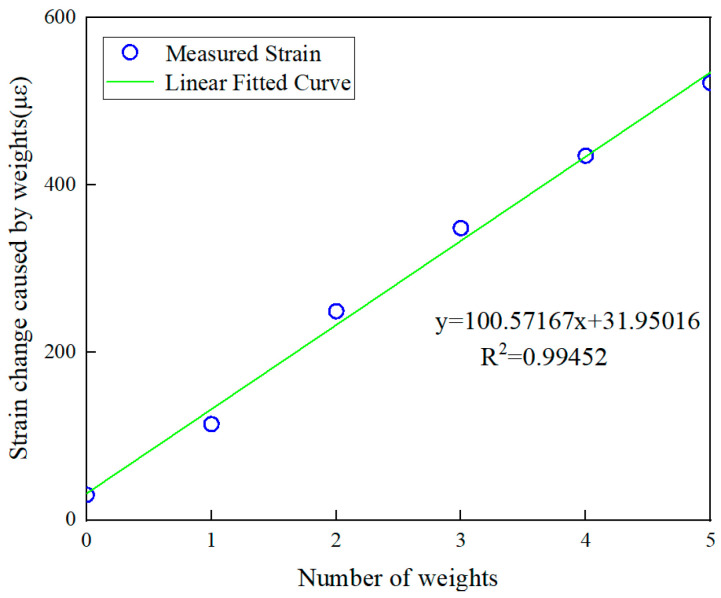
Measured strain data using traditional strain gauge.

Based on the simulation results, the working frequency in this paper is selected within the range of 2~4 GHz, and the VNA is calibrated by using the mechanical calibration piece ZN-Z135 from Rohde & Schwarz (Munich, Germany), which is typical of this range. Figure 14 shows the measured strain of traditional metal foil strain gauges. Before conducting strain testing on metal foil strain gauges under different weights, temperature compensation and balance zeroing operations were performed to ensure the accuracy of the data. It can be seen that the maximum strain does not exceed 600 με after adding five weights. On this basis, the sampling frequency range was determined to be 10 MHz to accurately capture the strain-derived frequency drift of the antenna., A total of 5001 points were taken within this range to obtain better recording results, meaning we used a sampling interval of 2 kHz between two sampling points. During the test, the five weights were added one by one, and the strain data of the metal foil gauge and the resonance frequency data of the VNA were recorded simultaneously.

### 4.2. Experimental Results and Discussion

**Figure 15:**
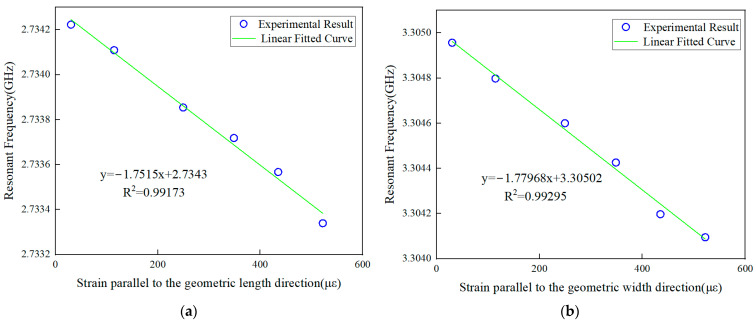
Relationship of strain–experimental resonance frequency in small strain range (**a**) along the geometric length direction and (**b**) along the geometric width direction.

As shown in Figure 15, value of horizontal axis represents the strain data measured by traditional metal foil strain gauges. Figure 15a is the strain monitoring results of the direction parallel to the length direction of the antenna, and Figure 15b is the monitored strain along the width direction. The strain sensing sensitivity parallel to the length direction is 1.715 kHz/με with a correlation coefficient of 0.99173, showing the good sensing linearity of the antenna. Moreover, the strain sensing sensitivity parallel to the width direction is 1.779 kHz/με, and the correlation coefficient is 0.99295. The good linear relationships between the resonant frequency drift and the strain in both directions are consistent with the above theoretical and simulation results. In addition, the strain sensing sensitivities obtained in the experiment is lower than that obtained in the simulation. The possible reasons for this are as follows.

(1) The microstrip line is not completely fixed to the equal-strength beam [4], and there may be slip between the microstrip line and the surface of the beam below.
(2) The antenna sensor is very sensitive to the surrounding environment [5]. The environmental noise is ignored in the simulation, but the environmental noise in the actual test will affect the experimental results.
(3) The antenna and the microstrip line rely on the conductive gel to conduct electricity, but its existence makes the contact between the antenna and the microstrip line insufficient.
(4) There is strain transmission loss between the beam surface and the antenna patch because the bonding layer and the substrate are polymer materials with viscoelasticity and a hysteresis effect [6]. In this case, the strain of the equal-strength beam cannot completely transfer to the antenna radiation patch, leading to a decline in sensitivity.
(5) During the simulation, vacuum conditions and a Perfect E surface were used, and the size of the antenna met our criteria [7]. However, in actual experiments, due to factors such as processing technology, testing environment, and instruments, there may be differences between the simulation results and those used.

In order to achieve accurate sensitivity in practical applications measuring actual strain, the following suggestions may be referenced. For instance, in order to minimize the influence of environmental factors such as temperature and humidity on measurements, compensation design can be carried out [8]. Next, simulation and experimental verification may be conducted in advance [7]. Additionally, it is necessary to perform calibration tests on experimental devices before the experiment.

## 5. Conclusions

This paper presents a flexible microstrip antenna based on graphene films for the simultaneous measurement of large strains in two perpendicular directions. The antenna’s dimensions were optimized through the application of Smith chart analysis and impedance matching principles. We identified the optimal impedance and feed line embedding positions within the 2–4 GHz frequency range, yielding an optimal set of antenna size schemes for impedance matching. Our simulations and experiments confirmed that the antenna’s initial resonant frequency is inversely proportional to its geometric size, and that strain sensing sensitivity is directly proportional to this initial resonant frequency. The designed rectangular microstrip antenna demonstrated a strong linear relationship between resonant frequency and applied strain in both lengthwise and widthwise directions, indicating its potential to accurately measure both minor and significant strain deformations. Future research will focus on the practical implementation of this antenna, including its integration into larger-scale systems and the exploration of its potential in various strain sensing applications.

## Figures and Tables

**Table 1:** Different feed position and the corresponding resistance axis intersection point value.

	*y* = 1	*y* = 2	*y* = 3
*x*_1_ = 1	1.10, 1.18	1.21, 1.21	1.24, -- ^a^
*x*_1_ = 2	1.12, 1.21	1.16, 1.16	1.21, --
*x*_1_ = 3	1.14, 1.28	1.24, 1.31	1.32, --
*x*_1_ = 4	1.14, 1.28	1.25, 1.31	1.31, --
*x*_1_ = 5	1.16, 1.73	1.27, 1.62	1.58, 1.69
*x*_1_ = 6	1.17, 1.99	1.24, 1.91	1.91, --
*x*_1_ = 7	1.12, 2.51	1.17, 2.38	1.67, 2.38

^a^ --: No corresponding intersection point, indicating poor impedance matching.

**Table 2:** Eccentric feed antenna size design scheme.

Parameter	Value (mm)	Parameter	Value (mm)
*w*_1_	25.00	*l*_1_	30.00
*w*	50.00	*l*	60.00
*x*	2.25	*y*	2.00
*x*_1_	2.00	*t*	1.25

**Table 3:** Strain sensing sensitivity values obtained from the simulation.

	Small Strain (0–600 µε)	Large Strain (0–65,000 µε)	Difference
Along width direction	3.194 kHz/με	3.293 kHz/με	3.01%
Along length direction	2.861 kHz/με	2.864 kHz/με	0.10%

## Data Availability

The data used to support the findings of this study are available from the corresponding author upon request.
